# Cytologic picture of Castleman’s disease: A report of two cases

**DOI:** 10.4103/0970-9371.73306

**Published:** 2010-10

**Authors:** Ayyagari Sudha, Namala Vivekanand

**Affiliations:** Consultant Pathologist, Medwin Hospitals, Hyderabad, India; 1 Department of Pathology, Osmania Medical College, Hyderabad, India

**Keywords:** Cytology, Castleman’s disease, lymph node, fine needle aspiration cytology

## Abstract

Castleman’s disease (CD), also called giant lymph nodal hyperplasia, is a lymphoproliferative disorder secondary to lymphoid follicle hyperplasia and marked capillary proliferation with endothelial hyperplasia. It presents as enlarged lymph nodes in the mediastinum, neck, groin, axilla and other sites. On clinical examination as well as gross examination, this disease mimics lymphomas and tuberculosis. Since cytological appearances vary depending on the type and extent of hyperplasia, fine needle aspiration cytology (FNAC) findings may not always be conclusive in all cases. We studied aspirates in two cases of CD, one of which presented with multiple enlarged axillary lymph nodes and the other with enlarged cervical lymph node. Cytology revealed reactive lymphadenitis with hyalinized capillaries and other features. Lymph node excision in both cases confirmed the diagnosis.

## Introduction

Castleman’s disease (CD; also known as giant lymph node hyperplasia, lymph nodal hamartoma) represents a morphologically distinct form of lymph node hyperplasia rather than a neoplasm or hamartoma. The etiology is unknown, the two main hypotheses being abnormal immune response and viral infection.[1] It usually presents as localized or systemic lymphadenopathy or even as extranodal mass and may give rise to several differential diagnoses.[2] There are two types, hyaline vascular and plasma cell types, but in general, histology is characteristic. Follicles are usually of the regressively transformed type rather than progressive transformation seen in reactive hyperplasia. We studied aspirates in two cases of lymphadenopathy (cervical and anterior axillary) in which the cytology was indicative of CD and which were subsequently confirmed by histology.

## Case Report

We studied fine needle aspiration cytology (FNAC) findings and subsequent histology of two cases, the details of which are given below.

### Case 1

A 38-year-old lady was referred for FNAC of multiple left axillary nodes, one of them in the axillary tail of left breast, which appeared as a breast lump in the upper outer quadrant. The nodes ranged from 1 to 3 cm in size, were well defined, mobile, firm and non-tender. There were no systemic symptoms and no other organomegaly. Peripheral blood counts and erythrocyte sedimentation rate (ESR) were within normal limits.

### Case 2

A 25-year-old man presented with a large, single left cervical lymph node, measuring 3 cm, since 3 months. Clinical diagnosis was hemangioma or paraganglioma. It was firm, mobile and non-tender. FNAC and subsequent histology of both the cases were similar.

### Cytology

**Figure 1:**
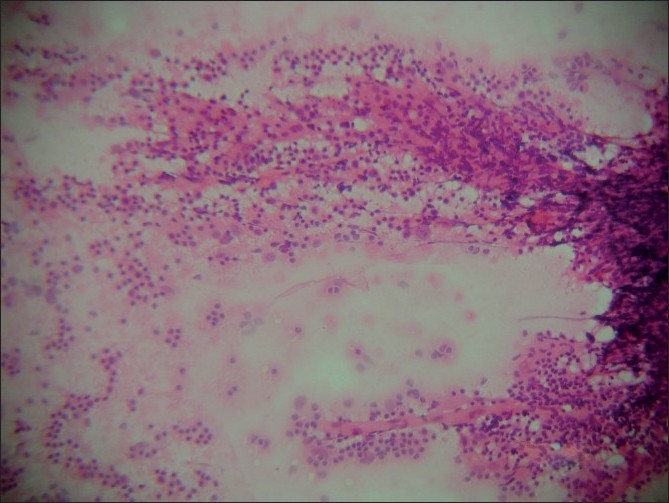
Smear shows hyalinized capillaries with adherent lymphoid cells in the aspirate (H and E, ×40)

**Figure 2:**
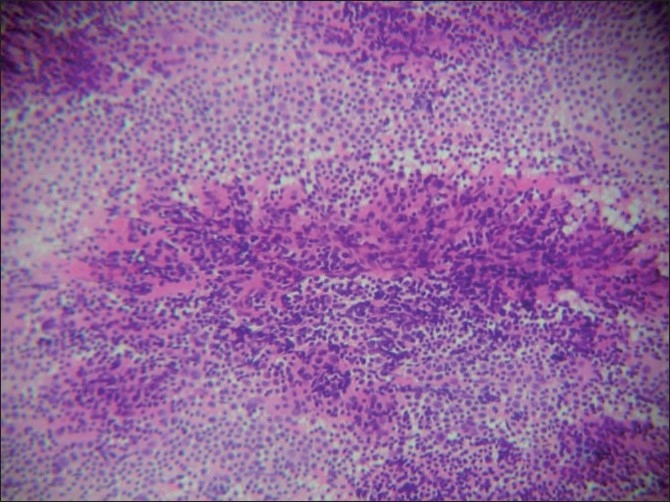
Smear shows granular eosinophilic material in the aspirate (H and E, ×40)

Smears were highly cellular and showed a mixed population of small and large lymphoid cells including follicular centre cells and tingible-body macrophages. There was prominent vascularity with hyalinized capillaries [Figure 1]. Germinal centre cells had eosinophilic material within the cell aggregates [Figure 2]. There were no epitheloid cells, giant cells or necrosis. Single, large abnormal cells were not significant; however, few immunoblasts were seen. Plasma cells were few in number. The cytological picture in both the cases was that of reactive hyperplasia with additional findings of hyalinized capillaries within germinal centre cells and eosinophilic granular material. A diagnosis of reactive hyperplasia with a strong indication of CD was made. Immunomarker study was not done on the aspirate since lymphoma was not suspected. Histopathology examination of the lymph nodes was advised in both the cases.

### Histopathology

**Figure 3:**
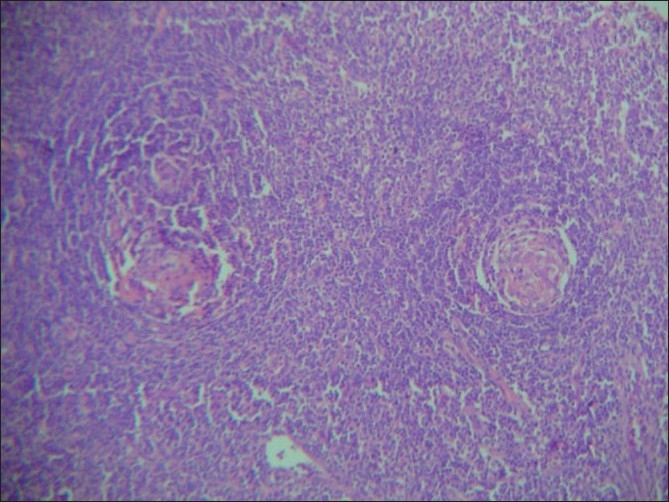
Section of lymph node containing vascularized germinal centres and eosinophilic material (H and E, ×10)

Grossly, the excised lymph nodes in both the cases were similar and measured 3×2.5×2 cm in size, were firm and had a grey tan with a homogenous cut surface. Microscopically, the capsule was thickened. Cortex and medulla contained many hyperplastic follicles of variable size, some of which were vascularized and had amorphous eosinophilic material. Para follicular zone contained branching capillaries, mature lymphocytes and plasma cells. Medullary sinuses were not seen. These findings confirmed CD, hyaline vascular type in both the cases [Figure 3].

## Discussion

The hyaline vascular type is the more common form of CD. The FNAC findings are those of follicular hyperplasia with mixed cells including lymphocytes, eosinophils, and immunoblasts as well as hyalinized capillaries. Follicles resembling Hassal’s corpuscles containing central hyalinized capillaries are reminiscent of lollipops. Occasionally, Reed Sternberg-like cells may be seen. In the plasma cell type, follicular hyperplasia, marked plasmacytosis with Russel bodies and occasional immunoblasts are characteristic.[3]

Large atypical follicular dendritic cells, considered dysplastic by some authors, were first described in association with CD in 1991. Awareness of the presence of dysplastic follicular dendritic cells within aspirates of CD will result in less cytological confusion in the future and may help to avoid the possibility of misdiagnosing Hodgkin’s lymphoma which has certain cytological similarities.[4] In the appropriate clinical context, a mature small lymphoid population associated with larger atypical cells which are consistent with follicular dendritic cells can be suggestive of CD.[5] However, we did not identify these dysplastic follicular dendritic cells, cells with crumpled tissue paper appearance and Reed Sternberg-like cells in our aspirates.

Frizzera *et al*.[6] studied clinical and histopathological findings in 15 patients of CD and opined that despite some similarities with autoimmune diseases, the main features of these process seem to best fit a hyperplastic–dysplastic lymphoid disorder in a setting of immunoregulatory deficit.

The possible pitfalls of this disease on cytology are missing the hyalinized capillaries with eosinophilic material in reactive hyperplasias and misdiagnosing single large cells and Reed Sternberg-like cells as Hodgkin’s lymphoma.

The differential diagnosis mostly includes all reactive hyperplasias of lymph nodes and, occasionally, Hodgkin’s lymphoma. Hsu *et al*.[7] demonstrated an abundant expression of IL-6 in most germinal centre B cells and in the numerous immunoblastic B cells in the mantle zone and interfollicular areas in the plasma cell variant of this disease, indicating the reason for increased plasma cell proliferation in lymph nodes and marrow as well as elevated gamma globulin level in the serum.

Hyaline-vascular CD is difficult to diagnose on FNAC and may be mistaken to be a lymphoreticular malignancy because of the presence of large cells having nuclei showing atypical features.[8] The cytomorphological findings in three histopathologically documented cases of hyaline-vascular CD were evaluated by Deschênes *et al*.[9] to a set of cytomorphological criteria that could help in the identification of this condition on aspirate smears. After review, the following cytomorphological criteria were suggested to be indicators of the lesion: (i) the presence of large oval to round cells having ill-defined cytoplasmic margins and large nuclei with irregular nuclear outlines having fine or coarse chromatin, giving a crumpled tissue paper appearance; (ii) a polymorphous population of lymphoid cells predominantly of small lymphocytes in the background. The authors concluded that although hyaline-vascular CD is a difficult diagnostic entity on aspirate material, the presence of large histiocytic cells with a crumpled tissue paper appearance of the nuclei in a background of small lymphocytes is a useful indicator for suspecting this lesion. However, these findings should be analyzed in larger studies to determine if they could in anyway reduce the diagnostic dilemma in cases of CD.[8]

Deschênes *et al*.[9] reported that the FNACs in three cases showed branching capillaries associated with fragments of germinal centre. A review of literature by them yielded 12 other case reports with over half describing similar findings. Because branching hyalinized small blood vessels penetrating follicular germinal centre are characteristic of CD of the hyaline vascular type on histology, this finding in fine needle aspirates should raise that diagnostic possibility.[9]

Our two cases also had this cytological finding. Though different authors have described many cytological findings and CD still remains a cytological dilemma, our FNAC smear findings in these two cases of CD helped us indicate a preoperative diagnosis thereby guiding the clinician in management of these patients.

## References

[1] Rosai J (2004). Rosai and Ackerman’s surgical pathology.

[2] Ghosh A, Pradhan SV, Talwar OP (2010). Castleman’s disease – hyaline vascular type – clinical, cytological and histological features with review of literature. Indian J Pathol Microbiol.

[3] DeMay RM (1996). The art and science of cytopathology.

[4] Taylor G.BG, Smeeton IW (2000). Cytologic demonstration of “dysplastic” follicular dendritic cells in a case of hyaline-vascular Castleman’s disease. Diagn Cytopathol.

[5] Meyer L, Gibbons D, Ashfaq R, Vuitch F, Saboorian MH (1999). Fine-needle aspiration findings in Castleman’s disease. Diagn Cytopathol.

[6] Frizzera G, Peterson BA, Bayrd ED, Goldman A (1985). A systemic lymphoproliferative disorder with morphologic features of Castleman’s disease: clinical findings and clinicopathologic correlations in 15 patients. J Clin Oncol.

[7] Hsu SM, Waldron JA, Xie SS, Barlogie B (1993). Expression of interleukin-6 in Castleman’s disease. Hum Pathol.

[8] Mallik MK, Kapila K, Das DK, Haji BE, Anim JT (2007). Cytomorphology of hyaline-vascular Castleman’s disease: a diagnostic challenge. Cytopathology.

[9] Deschênes M, Michel RP, Tabah R, Auger M (2008). Fine-needle aspiration cytology of Castleman’s disease:case report with review of the literature. Diagn Cytopathol.

